# Monitoring Progress towards Universal Health Coverage at Country and Global Levels

**DOI:** 10.1371/journal.pmed.1001731

**Published:** 2014-09-22

**Authors:** Ties Boerma, Patrick Eozenou, David Evans, Tim Evans, Marie-Paule Kieny, Adam Wagstaff

**Affiliations:** 1 World Health Organization, Geneva, Switzerland; 2 World Bank Group, Washington, D.C., United States of America

## Abstract

Universal health coverage (UHC) has been defined as the desired outcome of health system performance whereby all people who need health services (promotion, prevention, treatment, rehabilitation, and palliation) receive them, without undue financial hardship. UHC has two interrelated components: the full spectrum of good-quality, essential health services according to need, and protection from financial hardship, including possible impoverishment, due to out-of-pocket payments for health services. Both components should benefit the entire population.

This paper summarizes the findings from 13 country case studies and five technical reviews, which were conducted as part of the development of a global framework for monitoring progress towards UHC.

The case studies show the relevance and feasibility of focusing UHC monitoring on two discrete components of health system performance: levels of coverage with health services and financial protection, with a focus on equity. These components link directly to the definition of UHC and measure the direct results of strategies and policies for UHC. The studies also show how UHC monitoring can be fully embedded in often existing, regular overall monitoring of health sector progress and performance. Several methodological and practical issues related to the monitoring of coverage of essential health services, financial protection, and equity, are highlighted. Addressing the gaps in the availability and quality of data required for monitoring progress towards UHC is critical in most countries.

## Abstract 2

As part of the Collection on Monitoring Universal Health Coverage, Ties Boerma and colleagues discuss the key findings from the country case studies and technical reviews included in the Collection and, also, how these papers will help with the development of a global framework for monitoring progress towards Universal Health Coverage.

*Please see later in the article for the Editors' Summary*

**Summary Points**

- The overall goal of universal health coverage (UHC) is that all people obtain the good-quality essential health services, including promotion, prevention, treatment, rehabilitation, and palliation, that they need without enduring financial hardship.
- A global UHC monitoring framework, developed by WHO and the World Bank Group in interaction with the process that led to this PLOS Collection, was used in 13 country case studies, underpinned by five technical reviews.
- The UHC monitoring framework focuses on the simultaneous monitoring of coverage of the population with essential health services and with financial protection against catastrophic out-of-pocket health payments, stratified by wealth quintile, place of residence, and sex.
- Most countries focus on regular monitoring of a set of tracer indicators for priority health services, as well as the occurrence of financial hardship and impoverishment due to out-of-pocket health expenses. The indicators generally follow international standards of measurement and can be used for global comparisons.
- Most countries do not have an explicit framework for UHC monitoring. The monitoring of UHC is, however, partially embedded in regular overall health sector progress and performance reviews which include health system inputs, service delivery, and health status indicators.
- There are major gaps in the availability and quality of data required for monitoring progress towards UHC. Countries mostly rely on international survey programs or national surveys to obtain disaggregated data on coverage and financial protection indicators, complemented by health facility data, but often the frequency and contents of these surveys are not sufficient to meet the country's information needs.
- Monitoring progress towards the two components of UHC will be complementary and critical to achieving desirable health outcome goals, such as ending preventable deaths and promoting healthy life expectancy, and also reducing poverty and protecting household incomes.

## Introduction

A movement towards universal health coverage (UHC)—ensuring that everyone who needs health services is able to get them, without undue financial hardship—has been growing across the globe [1]. Close to half of the countries of the world—across all income levels—are currently engaged in health reforms that aim to extend, deepen, or otherwise improve coverage with needed health services and/or financial protection. These reforms have led to a sharp increase in the demand for expertise, evidence, and measures of progress and also a push to make UHC one of the goals of the post-2015 development agenda [2].

UHC has been defined as the desired outcome of health system performance, whereby all people who need health services (promotion, prevention, treatment, rehabilitation, and palliation) receive them, without undue financial hardship [1]. UHC has two interrelated components: the full spectrum of good-quality, essential health services according to need and protection from financial hardship, including possible impoverishment due to out-of-pocket payments for health services. Both components should benefit the entire population. In the context of this framework, “essential” is used to describe the services that a country decides should be available immediately to all people who need them. The contents of the services vary by setting.

**Figure 1:**
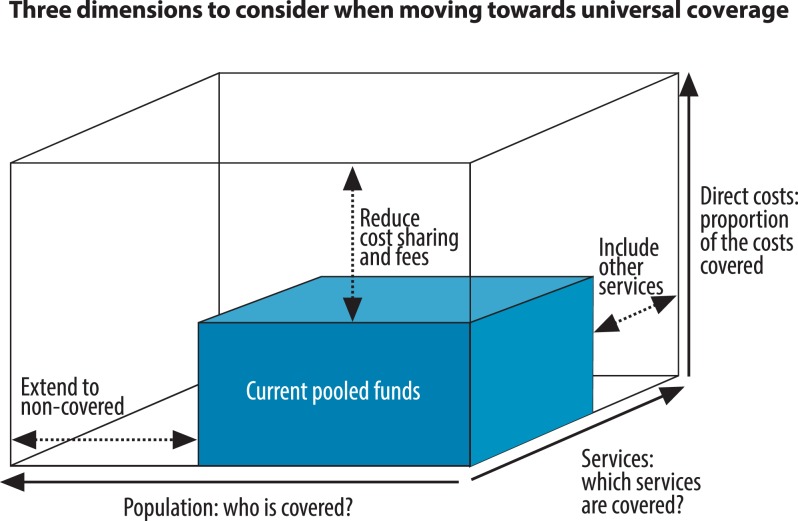
Progressive realization of universal health coverage.

The dimensions have commonly been depicted as a cube, shown in Figure 1 (adapted from [3],[4]). The first axis represents the population, the people who need health services. The services axis depicts the quality health services they need. The vertical axis is the proportion of the total cost of providing services to the population that is financed through “pooled financing systems” as opposed to direct payments by patients, shown in Figure 1 as the box labelled “current pooled funds.”

In this illustration, a little more than a half the population is covered for about half of the possible services they need, but only half the cost of these services is met from pooled funds. There is thus a shortfall of service coverage among those who receive services, inequity in service coverage (a large fraction of the population receives no services), and a lack of financial protection (those who receive services pay a large part out-of-pocket and hence risk financial hardship). To get closer to UHC, the country would need to provide services to the people who currently need them but don't receive any, provide more services to those who currently receive some but not the full range of services they need, and raise the fraction of health spending financed through pooled funds to improve financial protection. At the same time, health services need to be of sufficient quality to achieve the desired outcomes, so improving quality will be a priority in many settings.

Each country progresses in filling the different dimensions of the box (Figure 1) according to its preferences and constraints, trading off what services are provided, who gets them, and how much they are financed out of pooled funds. As such, UHC is the ultimate objective or goal, with countries starting from different places, with very different health problems, health systems, and resources. They need to find their own paths.

UHC is a dynamic, rather than static, concept. New health technologies and medical products are developed continually, as are new ways of improving the quality of care. The health service axis in Figure 1, therefore, expands over time. Many of the innovations come at higher costs, while population demands for new and better technologies also increase, putting upward pressure on pooled resources and making it harder to hold constant—let alone raise—the share of spending financed through pooled resources. That is why the search to attain and maintain UHC concerns even the richest countries, particularly at times of financial crises, when their ability to maintain high standards of service coverage and low household out-of-pocket payments is put under considerable strain.

This PLOS Collection focuses on the monitoring of progress towards UHC, which should be a central component of any UHC strategy. Country case studies and technical reviews were conducted as part of the development of a global monitoring framework by World Health Organization (WHO) and the World Bank Group. The country case studies [5]–[17] aimed to document what indicators, measurement, and communication approaches work best to monitor progress towards UHC. The technical review papers addressed issues related to the measurement of financial protection [18], service coverage [19], effective coverage [20], equity and UHC [21], and as an example of a health program, the implications for tuberculosis program monitoring [22].

The second version of the WHO/World Bank Group UHC monitoring framework was published in May 2014 [23]. In addition to the country case studies and technical reviews, the framework was based on consultations and discussions with country representatives, technical experts, and global health and development partners [24]. The feedback and country case studies were synthesized and reviewed at a meeting of country and global experts in Bellagio, Italy, in March 2014 [25]. The framework was modified to reflect the views emerging from these consultations and lessons learned from the country case studies.

The UHC monitoring framework aims to inform and guide assessment of both aggregate and equitable coverage of essential health services as well as financial protection. Monitoring progress towards these two components of UHC will be complementary and critical to achieving desirable health outcome goals, such as ending preventable deaths and promoting longer healthy life expectancy, and also reducing poverty and protecting household incomes. The main characteristics of the monitoring framework are described in Box 1. The global goal and proposed targets and indicators are presented in Box 2.

**Box 1. Framework for Monitoring Universal Health Coverage: Key Characteristics [23]**

Monitoring universal health coverage (UHC) in countries is **part of the regular system** of health progress reviews and systems performance assessment of the national health sector strategic plan, which includes tracking trends and inequalities in health system inputs and outputs, coverage and risk factors, and health outcomes.

The UHC monitoring framework focuses on **two interrelated but separate measures:** coverage of the population with essential health services and coverage of the population with financial protection against catastrophic out-of-pocket health payments. Progress on both measures should be measured simultaneously and capture all levels of the health system. Some interventions, such as tobacco taxes, are society-wide, while others, such as emergency obstetric care, are provided in health facilities. Similarly, financial protection measures should cover all levels of the health system, as costs incurred for services may vary widely.

All measures should be **disaggregated** by socioeconomic and demographic characteristics where relevant in order to allow assessment of the equitable distribution of service and financial protection coverage. In all health systems, there is significant stratification of risks for ill-health and access to and payments for services according to household income, place of residence, sex and other factors. Measures of coverage with health services and financial protection should also benefit the entire population throughout the life-course, including all ages and both sexes.

Measures of service coverage comprise the **full spectrum of essential health interventions**—promotion, prevention, treatment, rehabilitation, and palliation—and their associated costs. Special attention should be given to the quality dimension of the interventions.

Countries should focus on regular monitoring of a set of **tracer indicators with targets** for a selected set of priority health services and the occurrence of financial hardship and impoverishment due to out-of-pocket health expenses. The indicators should follow common standards of measurement and include global measures.

While countries develop indicators and targets in line with their level of socioeconomic development, epidemiological situation, state of the health system and people's expectations, there should also be a small set of global measures and targets that is **relevant to all countries**, irrespective of their national income (see Box 2 for proposed indicators).

**Box 2. Proposed Goal, Targets, and Illustrative Indicators for UHC in the Global Framework [23]**

**Goal**

•Achieve UHC. All people obtain the good-quality essential health services that they need without enduring financial hardship.

**Targets**

- By 2030, all populations, independent of household income, expenditure or wealth, place of residence, or sex, have at a minimum 80% essential health services coverage.
- By 2030, everyone has 100% financial protection from out-of-pocket payments for health services.

**Indicators**

***Health services coverage***

- *Prevention:* coverage with a set of tracer interventions for prevention services (see [19] for examples).
  1. *Equity:* a measure of prevention service coverage as described above, stratified by wealth quintile, place of residence, and sex.
- *Treatment*
  1. *Aggregate:* coverage with a set of tracer interventions for treatment services (see [19] for examples).
  2. *Equity:* a measure of treatment service coverage as described above, stratified by wealth quintile, place of residence, and sex.

***Financial protection coverage***

- *Impoverishing expenditure*
  1. *Aggregate:* fraction of the population protected against impoverishment by out-of-pocket health expenditures, comprising two types of household: families already below the poverty line on the basis of their consumption and who incur out-of-pocket health expenditures that push them deeper into poverty; and families for whom out-of-pocket spending pushes them below the poverty line.
  2. *Equity:* fraction of households protected against impoverishment or further impoverishment by out-of-pocket health expenditures, stratified by wealth quintile, place of residence, and sex.
- *Catastrophic expenditure*
  1. *Aggregate:* fraction of households protected from incurring catastrophic out-of-pocket health expenditure.
  2. *Equity:* fraction of households protected from incurring catastrophic out-of-pocket health expenditure stratified by wealth quintile, place of residence, and sex.

This overview of the PLOS Monitoring Universal Health Coverage Collection synthesizes selected findings of the country case studies and technical reviews, considering key topics such as the implications of the diversity of UHC policies and strategies for country monitoring frameworks, approaches to monitor health service coverage, financial protection and equity, the use of targets and summary measures, and the required investments to improve monitoring. The paper concludes with the way forward related to global guidance and country implementation, indicators and targets, and measurement investments.

## Country Monitoring

Monitoring progress towards UHC by countries should take into account the country's unique epidemiological and demographic profile, health system, level of economic development, and the population's demands and expectations. These country-specific dimensions are critical for deciding what should be monitored; for example, emerging economies might focus on how best to expand essential services to remote areas, whereas high-income countries might focus on modifying the range of available health services to allow for a growing elderly population. While the country context determines the measures used, the domains to be monitored—coverage with essential, good-quality services and with financial protection—are relevant to all countries, regardless of their level of income, their demographic profile or their health needs.

The PLOS Collection country case studies show the variation of ways in which UHC has been reflected in national policies and strategies. Often UHC policies build upon other policies that have been in existence for many years (e.g., Estonia [10], Ghana [11], and India [12]). Several countries have well-established strategies to increase access, coverage, and quality of services and financial protection among all population groups, such as Thailand [16], Brazil [6], Singapore [13], and Chile [7]. Some countries have linked the UHC goal with broad health reforms (e.g., China [8], Estonia [10], and Ghana [11]). Other countries are in the process of developing overall UHC policies and focused largely on the Millennium Development Goals (MDGs), but are also expanding strategies to enhance access to services and financial protection (e.g., Ethiopia [9], Bangladesh [5], and Tanzania [15]).

No matter what policy or strategy is in effect or the stage of implementation, countries will have to embed UHC progress monitoring in overall monitoring of health system performance and health progress. Currently, no country has a separate monitoring framework for UHC, but for some, UHC is well integrated in the overall sector monitoring framework (e.g., Thailand [16] and Estonia [10]). The experience from Thailand [16] describes how a system that monitors progress towards UHC can be built up over a long period, but having a solid framework with indicators, targets, data sources, data quality assessment and analysis, and clear roles and responsibilities of country institutions is likely to improve monitoring and enhance its efficiency.

The indicators used to monitor health sector performance generally include the main UHC progress indicators. For instance, Ethiopia already monitors three dozen service coverage and financial protection indicators on a regular basis [9]. Furthermore, in other countries the assessment of the current situation and recent progress towards UHC includes a focus on coverage and financial protection indicators, but also considers the full array of health system performance indicators at the same time (e.g., Chile [7] and Estonia [10]). In Singapore, even though there is no monitoring framework for UHC, indicators of access, quality, and affordability of services are regularly tracked and reported to Parliament as part of the key performance indicators of the Ministry of Health [13]. Finally, several countries have extensive systems of periodic health sector performance reviews at subnational and national levels which provide an excellent vehicle for UHC monitoring (e.g., Ghana [11] and Brazil [6]).

The paper on monitoring UHC in the context of tuberculosis care and prevention provides a global example of how the disease-specific monitoring of intervention coverage and financial protection take into account the full array of indicators, from input to impact, to assess programme performance [22].

### Coverage of Health Services

Measures for monitoring specific health interventions and reductions in risk factors can be classified differently, depending on the condition, the type of intervention, the characteristics of the target population, and the level of delivery of the intervention. In the UHC monitoring framework, the measures are grouped into two broad categories to cover the spectrum of interventions: prevention (which includes services for health promotion and prevention) and treatment (which includes services such as treatment, rehabilitation, and palliation). There are many service coverage indicators. Drawing on indicators agreed on by WHO for monitoring intervention coverage in the context of the MDGs and noncommunicable diseases (NCDs), the framework proposes measurement of coverage for a small set of prevention and treatment tracer interventions based on criteria related to relevance, quality, and availability of the indicators [19]. This core set of interventions can be built upon over time as and when comparable, reliable measures of coverage for other intervention areas, such as rehabilitation and palliation, become available.

The country case studies clearly show that in several countries, such as Bangladesh [5], Ethiopia [9], Ghana [11], and Tanzania [15], monitoring progress is focused on the MDG-related indicators, mostly with data from internationally comparable household survey programmes such as Demographic and Health Surveys. Other countries focus much more on noncommunicable disease–related interventions, such as Brazil [6], Chile [7], Singapore [13], and Thailand [16], which is largely done through specific national surveys and facility data. While the differences in epidemiology are an important contributor to those differences, a comprehensive health information system will be needed in all countries in the future to monitor progress towards UHC.

The relative paucity of good indicators of treatment coverage in the country case studies reflects the difficulty in determining needs for conditions that affect only a fraction of the population and often require facility-based care, such as cancer treatment or appendectomy. This lack of data on true population need is an important concern, as illnesses that require hospitalization or long-term treatment are often associated with higher financial risks, and many people may forgo these services because they cannot afford them. Even in high-income countries for which there are extensive data, very few treatment coverage indicators are in routine use [19],[26]. Nevertheless, for conditions with reliable and valid methods to determine population need, such as biomarkers for hypertension or diabetes, household surveys can help determine the size of the population in need and also the number treated.

In circumstances where treatment coverage is difficult to measure, disaggregating general service utilization rates by equity stratifiers offers a proxy for UHC monitoring. For example, in Chile [7] and Brazil [6], monitoring of secondary and tertiary care intervention rates by wealth quintiles showed that the poorest quintile had much lower intervention rates compared with the wealthiest quintile. Such data are useful for monitoring UHC, but often need additional analyses to account for the differences in need among populations.

UHC coverage monitoring should not only take into account the need and utilization but also the quality of the service, often referred to as “effective coverage” [20]. Effective coverage indicators capture all three components of coverage. The paper by Ng et al. [20] reviews different conceptual aspects and potential methods for the measurement of need, utilization and quality of services. Using examples from research, it is shown that the tracking of effective coverage for most interventions is dependent on a well-developed surveillance system to allow triangulation of health information that captures both demand and data.

### Coverage of Financial Protection

Existing measures of financial protection provide useful insights into the financial hardship caused by accessing needed health services. The paper by Saksena et al. [18] reviews four indicators of the lack of financial protection to show average levels and inequalities on the path to UHC.

Two commonly used indicators to track the level of financial protection in health are the incidence of “catastrophic” health expenditures and the incidence of impoverishment because of out-of-pocket health payments [18]. The former indicates the number of households of all income levels that incur health payments that are higher than their resources, while the latter captures the degree to which health spending causes extreme hardship by pushing families below the poverty line. The two financial protection measures actually measure lack of financial protection in health, and both can be re-scaled so that 100% coverage represents full financial protection [18].

Two other indicators that are sometimes used, although they are less understandable and accessible to policy-makers, are “depth of poverty,” the extent to which out-of-pocket health payments worsen a household's pre-existing level of poverty, and the “mean catastrophic positive overshoot,” the average amount by which households affected by catastrophic expenditures pay more than the threshold used to define catastrophic health spending.

The impoverishment indicator does not capture families that are pushed even further into poverty by out-of-pocket health spending; a simple way to capture this value is to add the number of non-poor families impoverished by health spending to the number of already poor families who incur out-of-pocket payments. The total is simply the number of households that are pushed into poverty, or deeper into poverty, because of health spending.

Most country case studies in this PLOS Collection also provide data on coverage of the existing insurance schemes in the general or target population(s) (e.g., Estonia [10], China [8], and Singapore [13]). Insurance coverage, however, is not used as a proxy for the above indicators of financial protection. For instance, the India case study shows how the multiple subsidized health insurance schemes for poor families fail to access the main sources of the out-of-pocket payments, which are to obtain ambulatory care and medicines.

The paper by Lönnroth et al. [22] argues that monitoring financial protection because of out-of-pocket health care expenditures is essential but will not guarantee effective and equitable tuberculosis (TB) care and prevention. Additional financial risks associated with tuberculosis include income losses from lost work and non-medical expenditures such as transport and food. The authors also explore ways to mitigate these losses.

### Equity in Coverage

At the heart of UHC is a commitment to equity. Yet, in countries on the path to UHC, there is a risk that poorer, less advantaged segments of the population could be left behind [27]. The global framework proposes three primary elements for disaggregation that can be measured comparably in all settings: household income, expenditure or wealth (coverage of the poorest segment of the population compared with richer segments), place of residence (rural or urban), and sex. The paper by Hosseinpoor et al. [21] provides a set of recommendations on global monitoring of health inequalities in the context of UHC, including the use of at least two complementary measures (such as wealth quintile, urban or rural residence, and sex where relevant), use of a gap or gradient analytical approach, and use of absolute or relative inequality as a measure of size of inequality gaps. Household surveys are often the prime instrument to collect data on equity [21], but facility data also contribute, particularly data on subnational differences [19]. For country monitoring of equity in coverage, the choice of stratifiers should be informed by an assessment of both those that are salient and those that are measureable, given the data available.

The PLOS Collection country case studies provide many examples of disaggregated monitoring of key coverage indicators. All countries use multiple stratifiers. In several countries, however, the subnational differences are of prime interest, because these are explicit in the national policy documents and are directly linked to budgetary decisions. For instance, Tunisia [17] and Ethiopia [9] focus on regional inequalities.

### Targets for Assessing Country Progress towards UHC

Setting specific, time-bound targets will be critical for progress towards UHC. Target setting will involve identifying from the available data sufficiently ambitious, yet achievable, improvements in equitable coverage of essential health services and financial protection.

The ultimate goal of UHC with respect to service coverage is that everyone can obtain the essential health services they need; that is, 100% coverage. The paper on service coverage argues, partly based on projections of trends in MDG service coverage indicators using different assumptions, that a minimum of 80% coverage, regardless of the level of wealth, place of residence or sex, is an ambitious, but nonetheless achievable, goal for most indicators and countries [19]. For some preventive services, such as vaccination coverage for specific antigens, higher targets are feasible based on current levels and past trends. But for most services, including full child immunization coverage, a minimum target short of the ideal may correspond better to the “sufficiently ambitious but nonetheless achievable” criterion. Targets must also include consideration of measurement issues. For some services, such as treatment of hypertension, effective coverage can reach 100% only if the treatment is 100% effective in achieving the desired health gain, which is rarely the case. Likewise, treatment indicators (such as for HIV infection) are often based on estimated need, which is rarely sufficiently accurate to set a target of 100%. Further analyses of time trends in coverage with prevention and treatment interventions and estimates of 2015 baseline and coverage improvement rates through to 2030 are required to further specify treatment coverage targets.

For financial protection, the available evidence suggests that a target that is both ambitious and achievable is 100% protection from both catastrophic and impoverishing health payments for the population as a whole as well as for the proposed equity strata of the population [18].

The rates of improvement necessary to achieve these targets in coverage over the next 15 years (to 2030) can be determined from the levels of coverage in 2015, with intermediate targets set for 2020 and 2025. The South Africa country case study in the PLOS Collection makes a case for setting UHC-related benchmarks for reduction of inequalities in service coverage and financial protection indicators as well as overall levels [14]. Such benchmarks could be country-specific but should also allow comparative analyses.

### Summary Measures

It is critical to communicate data on progress towards UHC in ways that are meaningful to the general public and that capture the attention of policy makers. One strategy is to focus on a small set of tracer indicators. Another is to use a summary measure of UHC progress. A third strategy is to use both tracer indicators and a summary measure.

Even though a summary measure will raise debate about weights for the different components, it may nonetheless be a useful way to communicate progress towards UHC. Summary measures, as simple and transparent as possible, should only be used if they help analyse, interpret, and communicate the situation and progress towards the goal of UHC.

Aggregation of measures entails an explicit approach to the criteria for weighting of interventions, which range from “equal” weighting (i.e., coverage with each service is given an equal weight); to “unequal” weighting, whereby the relative weight for the coverage of an intervention is affected by the size of the effect on mortality and morbidity, including both what has been achieved already and what potentially could be added if coverage was higher.

The Tanzania case study in the PLOS Collection includes a UHC access index that combines service coverage indicators with supply side indicators such as facility density or drug availability [15]. The intervention coverage paper in the PLOS Collection provides an example of prevention and a treatment summary measure based on a small number of tracer indicators that gives equal weight to intervention areas [19], similar to an approach used in monitoring equity in coverage of maternal, newborn, and child health interventions [28]. Attempts to combine health service access and financial protection into a single summary measure are, to-date, largely from a theoretical perspective [29].

The PLOS Collection shows how comparable data from four countries on 12 service coverage indicators can be used to compare the situations in a way that includes both the values of the individual interventions as well as an overall mean for the prevention and treatment domains [19]. For prevention services, six coverage indicators are identified: fulfilment of family planning requirements, at least four antenatal care visits, full immunization in children, improved water source and adequate sanitation, and non-use of tobacco. For treatment services, another six coverage indicators are used: skilled birth attendance, antiretroviral therapy, tuberculosis case detection and treatment success (combined into a single measure), hypertension treatment, and diabetes treatment.

### Investments in Data Collection

Regular monitoring of progress towards UHC requires reliable data on the selected indicators. Such data is obtained from household surveys and health facility data for service coverage indicators [19] and requires a well-functioning system of health accounts for the financial protection indicators [18]. Equity data are primarily obtained from regular household surveys, but facility and administrative data can be used to highlight trends and differences between geographic areas [21].

The PLOS Collection country case studies provide examples of the required investments and data gaps. Several papers point to the major data gaps for coverage of interventions and risk factors for NCDs and injuries (e.g., Tunisia [17] and Bangladesh [5]), even though these conditions are increasingly important in all countries.

Most country case studies relied heavily on household surveys, partly because these are the most objective source of population-based coverage and financial protection, but also because facility data–based systems often produce unreliable statistics. But there are also important gaps in the household survey implementation and contents. The reliance on internationally funded household health surveys often implies a focus on MDG-related indicators, while the country is facing a rapidly increasing burden of NCDs and injuries at the same time (e.g. Tunisia [17]). Many countries have some kind of household expenditure survey but do not have regular surveys to collect data on health-related household expenses. The value of a regular household survey that provides comprehensive and disaggregated information on service coverage and financial protection is shown in some countries, such as the five-yearly national health services survey in China [8]. Also, in India [12], regular national and district surveys are considered necessary to assess the financial risks associated with use of the private sector. The country case studies also show the potential value of investing in other regular data collection efforts such as surveys to assess user satisfaction, as was done in, for instance, Estonia [10].

## Discussion

The country studies and technical papers in this PLOS Collection show the usefulness and feasibility of the WHO/World Bank Group UHC monitoring framework. They illustrate how the framework can be used to translate the goal of UHC into measures of progress that are valid and feasible, and often comparable among countries. Together, these measures can provide a snapshot of health system performance with respect to coverage with some essential health services and financial protection, for the population as a whole and for critical subpopulations, based on household income, expenditure or wealth, place of residence, and sex. Using the targets and indicators, countries can identify their gaps in coverage and ascertain how far and fast they should improve the performance of their health systems to achieve progress towards UHC.

At this point in time, however, very few countries have an explicit Monitoring and Evaluation (M&E) framework for UHC at this stage. Such a UHC monitoring framework should be fully integrated in the existing overall health sector performance framework.

The global framework for monitoring UHC is designed to facilitate comparison of progress towards UHC among countries. Each country is expected to add further measures of service coverage and further equity stratifiers in order to tailor UHC monitoring to its context. UHC monitoring is not a substitute for other measures of health system performance, such as improved health status or health worker density and distribution. Rather, it should be seen as a core part of a comprehensive monitoring framework in which inputs are linked to outputs and health outcomes. The UHC measures of intervention coverage and financial protection can thus make a valuable contribution to assessment of health systems performance and to the achievement of desired health outcomes.

UHC is not built around a single universal package of interventions: each country should define the “essential” services that need to be available to all people. Therefore, the monitoring framework does not provide a single set of essential service indicators. However, the country case studies show that there is considerable agreement between the selected indicators. These indicators generally include prevention and primary care coverage indicators such as those related to the MDGs, including skilled birth attendance and antiretroviral therapy coverage, and the prevention and control of risk factors for NCDs. Only a few country studies included indicators of more advanced secondary and tertiary care interventions. These differences are partly associated with variations between countries in levels of socioeconomic development, health systems, and epidemiological situations, which in turn affects the current priorities for the country's UHC strategy. But it is also because of limitations of the available data for the indicators.

The reviews of country UHC monitoring and global methodological issues show that there are a limited number of indicators of service coverage that are relevant, of reasonable quality, and feasible to measure with existing instruments, especially for the coverage of treatment services. Tracking of progress in financial protection measures is also hampered by lack of data. Investments are required to develop methods for devising a more comprehensive set of UHC indicators. Moreover, investing in data collection through household surveys using standardized questions and health facilities information systems is an important global public benefit and good value for the money in the pursuit of the goal of UHC. Only countries with a regular health examination survey on the main health priorities and a well-functioning health facility information system are able to generate the full set of disaggregated information on progress towards UHC.

Monitoring progress towards UHC is central to achieving the global goals of the World Bank Group and WHO, the MDGs, and the emerging post-2015 global development framework. The World Bank Group has set a global goal of ending extreme poverty by 2030. UHC is critical to achieving this goal, as it will prevent impoverishment of hundreds of millions of families because of out-of-pocket payments for health services. WHO places the highest priority on securing the right to health and attaining the highest levels of health for all. UHC secures universal entitlement to essential health services, which are important contributors to improving the health status of the population in all countries. Similarly, the World Bank Group's global goal to promote shared prosperity for the poorest 40% of the population in every low- and middle-income country is closely aligned with WHO's focus on equity and the United Nations High-level Panel's recommendation to “hardwire” equity into all post-2015 measures [2].

There is emerging consensus that the post-2015 agenda should address the unfinished agenda of the health-related MDGs as well as the emerging burden of NCDs, including mental health and injuries. There is already a strong foundation of health indicators to build upon, including the intervention coverage indicators [30] of the health-related MDGs, such as vaccination and antiretroviral therapy coverage, the recommended priority interventions related to NCDs [31],[32] and indicators of financial protection [33]. Further work needs to be done in consultation with countries and partners to identify and define specific prevention and treatment indicators. The importance of multisectoral influences on health should also be acknowledged, although it is not explicitly addressed in this paper. Further work is also needed to firmly link monitoring of progress towards UHC with monitoring of key social and environmental determinants of health and sustainable development. Further research and investments are needed to address these multiple information gaps, which should be a priority for research in the coming years [34].

## Conclusion

Based on this PLOS Collection it can be concluded that the global UHC monitoring framework provides an excellent basis for global and country monitoring of UHC, with appropriate adaptations. The focus is on country monitoring. Each country should develop a UHC monitoring framework, embedded in regular overall health progress and performance reviews that already exist in most countries. A monitoring framework includes the selection of a set of tracer indicators and targets, based on country demographic and epidemiological profile, health systems, level of socioeconomic development, and people's needs and expectations. Inequalities should be monitored across multiple stratifiers, including socioeconomic status, place of residence, and sex, where relevant. Monitoring the quality of services should receive special attention. International comparisons using tracer indicators can be used to benchmark country progress, keeping in mind the considerable diversity of UHC strategies and contents. Box 3 summarizes these recommendations.

**Box 3. Recommendations**

- The global UHC monitoring framework recommends the use of a set of tracer indicators of intervention coverage and financial protection, disaggregated by socioeconomic status, place of residence, and sex where possible and relevant.
- The core set of indicators and targets should be based on country demographic and epidemiological profile, health systems, level of socioeconomic development, and people's needs and expectations, and as a minimum, include a small set of globally recommended tracer indicators.
- Monitoring UHC should be fully embedded in regular overall health progress and performance reviews that exist in most countries.
- Countries and global partners should address data gaps and invest in comprehensive health information systems with regular health examination surveys that address the full burden of disease, surveys with health expenditure modules, and well-functioning health facility data reporting systems.
- International comparisons can be used to benchmark country progress, keeping in mind the considerable diversity of UHC strategies and contents.

To achieve such monitoring systems in countries, it is necessary to address data gaps and invest in comprehensive health information systems that include regular health examination surveys that address the full burden of disease, socioeconomic surveys with health expenditure modules, and well-functioning health facility data reporting systems. The global community can contribute by developing common standards for measurement of the core indicators, aligning and minimizing disease-specific monitoring efforts, and streamlining health data collection investments in support of national monitoring systems that meet all country needs.
